# Biomarkers Predicting Progression of Human Immunodeficiency Virus-Related Disease

**DOI:** 10.4021/jocmr2010.03.255w

**Published:** 2010-03-11

**Authors:** Amar Kanekar

**Affiliations:** Department of Health Studies, 200 Prospect Street, Denike 14 B, East Stroudsburg University of Pennsylvania, East Stroudsburg, PA 18301-2999, USA. Email: akanekar@po-box.esu.edu

## Abstract

Biomarkers in predicting the progression of HIV infected individuals to a state of HIV disease (AIDS) are studied over more than a decade. Use of surrogate markers in the past for tracking clinical progression of the disease was limited, as little knowledge existed about the disease. The aim of this review was to address various changes in biomarker related studies taking place over the last five years, especially the trend towards use of newer biomarkers and experimentation with novel molecules in a quest for halting HIV disease progression. An open search of PUBMED database was made with search 'key words' such as 'Biomarkers' and 'AIDS (Acquired Immunodeficiency Syndrome)'.The following were the inclusion criteria for articles: a) all articles published in English language, b) years of publication between 2002-2008 and c) articles limited to adult population. This yielded a total of 417 articles. The criteria used for further judging these studies considered a) type of research design, b) number of biomarkers studied, c) validity of the biomarkers, d) techniques to assess the biomarkers and the impact of the studies in furthering biomarker research, e) sample size for the studies and f) article title or abstracts having the following key words 'biomarker' or 'biomarkers' and 'predict progression to AIDS'. A total of 27 abstracts were reviewed and 12 studies met the above criteria. These 12 different studies consisted of three reviews, four cohort designs, three cross-sectional designs, one each of an observational, and an in-vitro design. The various biomarkers emerging as a results were primarily a mix of viral, neural, immunological, HLA (human leukocyte antigen) markers along with lymphocyte counts. Although there have been quite a few advancements in biomarker-related studies, majority of the novel biomarkers discovered need to be further evaluated and replicated in bigger, long-term efficacy trials. Efforts should also be made to discover newer genetic markers of disease progression. Biomarker feedback, a new concept, can be utilized in future studies addressing prevention of HIV infection or halting disease progression.

**Keywords:**

Biomarkers; Progression; Designs; HIV; AIDS; Validity

## Introduction

Acquired immunodeficiency syndrome or AIDS consists of a constellation of symptoms which are suggestive of end-stages of human immunodeficiency virus (HIV) infection. This syndrome involves loss or decrease in immunity against certain non-threatening illnesses. The HIV infects certain cells of the immune system and can also directly infect brain [1]. Infected individuals are called HIV positive individuals but without AIDS. Most individuals progress from this state towards the disease (AIDS) [2].

The pathogenesis of HIV infection involves series of dynamic interactions between the HIV virus and the host immune cells, which results in a state of continuous immune activation throughout the course of the infection [3]. To assess the biomarkers related to AIDS, we need to understand the spectrum of this disease, i.e., from getting infected with the HIV virus to developing full blown AIDS. A review article which looked at the immunological markers and surrogate markers for predicting clinical progression from HIV infection to AIDS, was able to discuss the importance of markers such as β_2_ microglobulin [4-8], neopterin [9-11], sIL-2R [12, 13], sCD8 [14, 15], antibodies such as anti-p24 [16, 17], antigp120 [18, 19], anti-p17 [20], anti-gp41 [21], anti-nef [22, 1], anti-sCD4 [23], and anti-leucocyte antibodies [24]. Some of the additional biomarkers studied in the past are antigen markers such as p24 antigens [25, 26], serological markers such as tumor necrosis factor α [27, 28], acid-labile human leukocyte IFN (interferons), 2-5A synthetase, percentage of CD4+ T-cells, absolute CD4+ T-cell numbers, and CD4+/CD8 T-cell ratio. It is clearly seen that there have been a plethora of biomarkers which have been studied which predict the progression of an HIV infected individual to a state of active disease of AIDS. Despite of having a large quantity of surrogate markers for the disease, their clinical use still remains debatable, as they fail to fulfill some of the important criteria like 1) having clear role in the natural history of HIV-induced illness, 2) being detectable in the majority of infected individuals, 3) changing measurably with clinical status in both progression and remission of disease and 4) changing quantifiably after a therapeutic intervention or no change following failure of therapy. Moreover, very few studies have shown the effect of treatment on surrogate markers and long-term survival. There is a great need for validation of these studies in larger trials before surrogate marker measurements would be accepted universally as clinical end-points [3].

Current research in the arena of biomarkers studies related to HIV/AIDS continues to be experimental and bereft of validated biomarkers. There is a shift in the cellular markers of disease progression from lymphocyte predominance to other cellular markers such as monocyte-macrophage system [29]. These preliminary experimental studies need to be followed by more longitudinal studies which discover newer biomarkers which are associative as well as predictive of progression of disease.

The following systematic literature review attempts to look at various studies conducted and published by HIV researchers related to use of biomarkers and its role in association or prediction of HIV infection or AIDS progression.

## Methodology

An open search of PUBMED database was made with search 'key words' such as 'Biomarkers' and 'AIDS (Acquired Immunodeficiency Syndrome)'. There were 2533 hits with this search. The following were the inclusion criteria for articles: a) all articles published in English language, b) years of publication between 2002-2008 and c) articles limited to the adult population. This yielded a total of 417 articles. The criteria used for further judging these studies considered a) type of research design, b) number of biomarkers studied, c) validity of the biomarkers, d) techniques to assess the biomarkers and the impact of the studies in furthering biomarker research, e) sample size for the studies and f) article title or abstracts having the following key words 'biomarker' or 'biomarkers' and 'predict progression to AIDS'. A total of 27 abstracts were reviewed and 12 studies met the above criteria.

**Figure 1.**
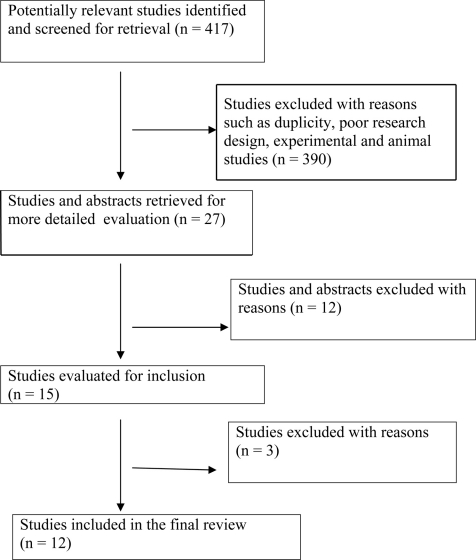
Progress through stages of systematic review of studies predicting progression of HIV virus related disease.

The Figure 1 is a summary of the study flow which states the progress through stages of the systematic review predicting progression of HIV virus related disease.

## Results

A summary of each study including the research design used, type of biomarkers studied the main outcomes of the study and relevant conclusions drawn are presented in Table 1. There were three review studies [40, 29, 43] which showed the importance of a) blood and cerebrospinal fluid markers such as CSF HIV 1 RNA and proviral HIV DNA, b) increase in macrophage makers and c) plasma viral HIV RNA, P24 antigen and CD4 cell counts, as biomarkers of progression of HIV infection. Three cross-sectional studies [31-33] looked at C-reactive protein and HLA (Human leukocyte antigens and soluble toll-like receptors as effective biomarkers of disease progression. This clearly shows the importance of immunological markers in the study of disease progression. There were four cohort studies [30, 34-36] which looked at immunoglobulin (IgG levels), neural markers such as sphingomyelin, TLC (total lymphocyte count), hemoglobin, and the CD4 cell counts to determine disease progression.

An observational study [41] used cox proportional hazards regression model to determine association between percent CD4 and disease progression while an in-vitro study [42] looked at regulatory T-cells as predictors of disease worsening. This study also showed a potential of developing new biomarkers by manipulating the regulatory T-cells.

**Table 1:** Biomarkers Studies Related to HIV Infection or Acquired Immunodeficiency Syndrome (Review Year 2002-2008)

Name of the study	Research design	Biomarkers	Results/discussion	Conclusions
1) Price et al (2007) [40]	Review	Viral: CSF HIV 1 RNA, neuropathogenetic genotypes Immunological: Beta 2 microglobulin,neopterin, quinolinic acid, MCP-1 Neural: molecular products of neurons, astrocytes, oligodendrocytes, microglia. Other: reactive aldehydes-4 hydroxy-nonenal	Elevated CSF HIV 1 RNA along with raised MCP-1 or neopterin suggest AIDS-dementia complex or HIV -1 encephalitis. Proviral HIV DNA within peripheral blood mononuclear cellsprognostic marker for ADC (AIDS-dementia complex). Monocytes showing increased expression of CD69, CD16, & TNF alpha.	Combination of blood and CSF markers should be used. Develop objective laboratory based quantitative measures for predicting and establishing diagnosis.
2) Gendelman (2007) [29]	Review	Quinolinic acid, chemokines, proinflammatory cytokines, matrix metalloproteinases surrogates as well as causal.	End-stage disease dementia and encephalitis occurs due to blood-borne macrophage entry in brain. Micro arrays done showed increase in RANTES increase in chronic stage.	Miroglial-macrophage activation underlies many divergent neurodegenerative disorders including neuroaids. There is further need of understanding host-defense as well as cell-signaling mechanisms.
3) Hulgan et al (2007) [41]	Observational study	Absolute CD4, percent CD4, and HIV 1-RNA	Used a cox proportional hazards regression model to determine associations between percent CD4 and disease progression. Prior ART (p < 0.0001), injection drug use (p < 0.04), lower absolute CD4 (p = 0.002), and lower percent CD4 (p = 0.002) predicted disease progression.	Percent CD4 at initiation of first HAART regimen predicted disease progression independent of absolute CD4. Percent CD4 may be used to determine timing of HAART (Highly active Antiretroviral therapy).
4) Nilsson et al (2006) [42]	In-vitro study	T-reg cell numbers(specialized subset of CD4 cells), FOX P3, CTLA-4, IDO, TGF B1(functional markers)	Increased number of FOX P3 positive T-cells and increased expression of T-reg cell associated markers were detected only during progressive HIV disease. T-reg cell exposure to HIV promoted their survival via CD4-gp 120 dependent pathway.	T-reg cells accumulate due to increase in HIV viral load. They negatively affect the immune control of virus replication. Potential novel ways of improving the immune function can be through manipulation of these T-reg cells.
5) Lau et al (2006) [32]	Cross-sectional study	C-reactive protein	The association of log10CRP(C-reactive protein) were inversely correlated with CD4 lymphocyte counts (r = -0.17, p < 0.001) and directly with log10HIV RNA levels (r = 0.20, p < 0.001). Levels of CRP of > 2.3 mg/l were associated with decreased time to AIDS (acquired immunodeficiency syndrome).	Levels of C-reactive protein were associated with HIV disease progression independent of CD4 lymphocyte counts and HIV RNA levels.
6) Kiepala et al (2005) [43]	Review	Viral markers: Plasma HIV RNA load, serum p24 antigen load, serum p 24 antibodies, syncitium inducing strains. Surrogate markers: Anti-p27 nef Immune markers: CD4 cell counts, percentage of CD4 cells, CD8 cell counts, neopterin, beta 2 micro globulin Other markers: CCR5, HLA type and resistance genotyping and phenotyping.	Plasma viral load (HIV RNA) is most representative and sensitive laboratory test as a predictor of risk for disease progression and response to antiretroviral therapy. P24 antigen was more specific, and cheaper but less sensitive. CD4 cell counts should be followed longitudinally and percentage of CD4 cells more accurately predicts progression.	Various statistical tests have indicated that CD4 cells, serum level of B2 microglobulin and p24 antigenemia in a descending order were best predictors of disease progression. Host genetic factors may be incorporated in assessment in the future.
7) Carbone et al (2004) [30]	Retrospective cohort study using cox proportional hazards model.	Immunoglobulin levels	High levels of soluble markers IgG (relative hazard (RH):1.06, p = 0.006), IgA (RH 1.67, p = 0.02), IgM (RH 1.28, p = 0.0001), B2-M (RH 2.38, p < 0.0001) and sTNF-R (RH 1.07, p = 0.002) individually showed progression to AIDS.	This is the first demonstration in a cohort of injection drug users that immunoglobulin level measurements have predictive value for HIV progression independent of CD4 T-cell counts and HIV RNA.
8) Heggelund et al (2004) [31]	Cross-sectional	sTLR 2- (soluble Toll-like 2 receptor) levels.	Chi-square analysis showed undetectable levels of sTLR 2 receptors in AIDS patients controlled with healthy controls (p = 0.02). No statistically significant correlation was found between sTLR levels and CD4 and CD8-T cell counts. HIV infected progressors to AIDS had decreased sTLR at all time points returned to baseline levels at last time point.	There is an association between sTLR levels and disease progression to AIDS. There can be an increased cytokine response to bacterial lipoproteins after sTLR levels depletion causing further progression of HIV disease.
9) Sacktor et al (2004) [34]	Cohort analysis	Sphingomyelin, ceramide, 2- pentylpyrrole lysine adduct.	There was an increase in two HNE (hydroxynonenal) adducts in medial frontal, cerebellum and cerebrospinal fluid of HIV dementia patients. Massive amounts of oxidative stress was seen with HIV encephalitis. Spingomyelins were raised in inactive dementia cases compared to HIV + cases without dementia; ceramides were elevated in active dementia cases.	Sphingomyelin metabolic products and markers of oxidative stress such as HNE and ceramide are elevated in actively progressive dementia cases. Surrogate markers must be correlated with clinical progression of HIV disease.
10) Fernandes et al (2003) [33]	Cross-sectional study	HLA-markers such as HLA- A1, A11, B8, B35, DR3, DR1, DQ2, DQ1, Bw4, B44, B57.	At least one of these HLA markers were exhibited by 56.4% of the patients associated with rapid progression to AIDS and 7.2% presented with at least one marker associated with slow progression to AIDS. The frequency of markers associated with rapid progression to AIDS was significantly increased in patients with CMV(cytomegalovirus) retinitis in relation to those without retinitis (P < 0.002).	HLA-markers associated with rapid progression to AIDS were significantly raised in CMV-retinitis group. HLA-markers may simultaneously predispose to AIDS and CMV-retinitis.
11) Lau et al (2003) [35]	Prospective cohort study	Total lymphocyte count and hemoglobin concentration	A total lymphocyte decline greater than 10% and Hgb decline greater than 2.2% was present in over 77% of HIV positive men who developed AIDS but only 23% of HIV positive who did not.	Both total lymphocyte count and hemoglobin concentration showed a period of stability which was followed by a rapid decline beginning before the onset of AIDS. Hence these markers are useful in monitoring disease progression in resource limited setting.
12) Jacobson et al (2002) [36]	Cohort study	CD4 lymphocyte cell counts.	Within 3.5 years of HAART (highly active antiretroviral therapy) initiation 11.3% of subjects developed AIDS and determinants of AIDS was a CD4 cell count of < 200cells/microl (relative hazard = 2.25 95% CI = 1.13, 4.49). An increase in CD4 counts of 50microlit. Immediately after HAART initiation also improved prognosis (RH = 0.34, 95% CI = 0.16, 0.71)	There is no difference between men who started HAART at a lower CD4 count and men who started HAART at a higher CD4 count from the time point of progression to AIDS. HIV RNA levels were good for prognosis but less informative in predicting death.

## Discussion

Biomarkers related to HIV infection or which predict the progress of HIV infection to AIDS are wide and varied. The trend in last 15 years in conducting biomarker studies has shown a shift from using immunological and surrogate markers such as β2-microglobulin [30, 5], neopterin [9, 10] and anti-leucocyte antibodies [24] to some newer markers such as sTLR2 (soluble toll-like receptors) [31], C-reactive protein [32] and HLA (human leukocyte antigen) markers [33]. There seems to be more research studies which are looking at neuropathogenic biomarkers predicting HIV infection of the central nervous system.

The biological basis for some of the newer markers (such as quinolinic acids, chemokines, and matrix metalloproteinases) in neuropathogenic HIV disease is surrogates as well as causal agents [29]. Looking at other important biomarkers, β-2 microglobulin and neopterin indicate the degree of immunological activation, CD4+ T-helper cells which have prognostic and diagnostic importance and HIV-specific antibodies whose decline is crucial in indicating prognosis of the disease [3]. The role of C-reactive protein in HIV pathogenesis is yet unclear [30] and rise in sphingomyelin, ceramides is due to oxidative stress which in turn is due to loss of cellular homeostasis in HIV infection [34].

There is a mix of cross-sectional, cohort and review studies which attempt to study biomarkers predicting progression of the disease. Some of these studies were associative studies and need replication in bigger trials. Validation of these various biomarkers is the need of the hour as some of the studies reviewed were experimental. The assessment of biomarkers were for predicting and establishing diagnosis of acquired immunodeficiency syndrome, understanding the pathogenic process of disease progression and developing newer genetic and surrogate markers for understanding the disease process. Surrogate marker usage in the past and at present continues, as they have a potential for reducing the cohort size required to conduct such studies and the duration of each trial.

Although biomarkers are useful in predicting the severity and progression of the disease, they should always be correlated with clinical disease progression. In developing world where access to advanced biomarker detection techniques is less, markers such as total lymphocyte count and hemoglobin concentration are useful in monitoring disease progression as shown in a prospective cohort study [35]. The past decade has seen extraordinary advancements in HIV therapy and with the advent of viral protease inhibitors in 1995, has invited multi-drug combination protocols such as highly-active anti-retroviral therapy (HAART). The effect of the HAART in changing the immunological profile of HIV infected individuals showed a decrease in absolute CD4, memory CD4 and nave CD4 lymphocytes while a prospective study showed no difference in time to AIDS progression when CD4 cells were used as the biomarkers. Although CD4 cells continue to be used as one of the best predictive biomarkers for disease progression, a suggestion of using HIV RNA is made from prognostic point of view [36].

The uses of genetic biomarkers for predicting progression of the disease shows mixed evidence. An international meta-analysis, which tried to assess the role of CCR5 delta 32 and CCR2-641 alleles on disease progression among pediatric population, showed no protection over long term [37] while another meta-analysis carried out in United States, Europe and Australia among adult population showed decreased risk of progression to AIDS and deaths [38]. Hence, it seems we need more studies to be fully convinced of the protective effects of these alleles in predicting HIV progression.

One of the ways of acquiring HIV infection is from indulging in unsafe sexual practices. It would be helpful for us as researchers, to know if biomarkers and biomarker feedback can be effective in generating healthy behavior change as a primary prevention strategy. Biomarker feedback has been used to study various behaviors such as smoking cessation, dietary change and increased physical activity with mixed results [39]. It can either make an individual motivated and getting ready for a behavioral change, or cause immense psychological distress and adverse behavioral changes. There is a need of additional research studies in these areas.

## Conclusions

In conclusion, the last decade has shown some advances in biomarker definitions and techniques for biomarker assays. These need to be validated over long-term by replicating studies in larger trials and with different population groups. Some of the biomarkers discovered are still in their nascent stages and may prove to be useful in future.
